# Therapeutic Notes

**Published:** 1893-01

**Authors:** David Cerna

**Affiliations:** Galveston; Demonstrator of Physiology in the Medical Department of the University of Texas; formerly Demonstrator of, and Lecturer on, Experimental Therapeutics in the University of Pennsylvania; Fellow of the College of Physicians of Philadelphia; Corresponding Fellow of the Sociedad Espanola de Higiene, of Madrid; Member of the Philadelphia County Medical Society; of the Pathological Society of Philadelphia; Associate Editor of Sajous’ Annual of the Universal Medical Sciences; Secretary (Section on Therapeutics) of the Pan-American Medical Congress, etc., etc.


### Therapeutics.

UNDER THE CHARGE OF DAVID CERNA, M. D., PH. D.,

Demonstrator of Physiology in the Medical Department of the University of Texas; formerly Demonstrator of, and Lecturer on, Experimental Therapeutics in the University of Pennsylvania; Fellow of the College of Physicians of Philadelphia; Corresponding Fellow of the Sociedad Espanola de Higiene, of Madrid; Member of the Philadelphia County Medical Society; of the Pathological Society of Philadelphia; Associate Editor of Sajous' Annual of the Universal Medical Sciences; Secretary (Section on Therapeutics) of the Pan-American Medical Congress, etc., etc.

PYOKTANIN IN THE TREATMENT OF CANCER.—Felix Baron von Oefele (*St. Petersburger Med. Wochens; Merch's Bulletin—La Revista Medico-Quirurgica*, October, 1892) has treated four cases of cancer with the above remedy. The author's method is as follows: To each patient he gives every morning, before breakfast, a capsule containing one milligramme ($\frac{1}{64}$ of a grain) of the phenate of cocaine, and nine centigrammes (1½ grains) of acetanilid (antifebrine); after an hour a light breakfast is allowed. Four hours afterwards, the author administers another capsule containing an alcoholic solution of pyoktanin, of the strength of 2%. This treatment is continued for four days; on the fifth day he orders one capsule of the pyoktanin, and a second dose, of the stated amount, four hours afterwards. Even a third capsule may be administered. The author affirms that under such a treatment patients notably improve; the pain disappears, the appetite returns, accompanied by an amelioration of the general strength and an increase of bodily weight.

THE TREATMENT OF CARBUNCLE.—The hypodermatic injection of carbolic acid, according to Alan B. Murray, of Cokedale, Montana (*N. Y. Medical Record—La Revista Medico-Quirurgica*,

October, 1892), has given good results in the treatment of carbuncle of the neck. The author relates three cases. He injected fifteen drops (0.9 gramme) of pure carbolic acid. The operation was but slightly painful, and as soon as the anæsthetic effects of the remedy were established, the infectious inflammation was changed into a simple circumscribed abscess, this being opened by incision in the course of a few days. The disease was thus considerably diminished in its duration.

---

GLYCERINE IN THE TREATMENT OF HEPATIC COLIC.—Ferrand (*Bulletin de la Academie de Medecine de Paris-Gaceta Medico-Quirurgica*, November, 1892) has published a good article on the above subject. The author draws the following important conclusions: 1. Glycerine does not dissolve hepatic calculi. 2. Administered by the mouth, in moderate quantities, the drug is rapidly absorbed, especially by the lacteals and the general lymphatic vessels. 3. In this manner, glycerine reaches the liver, and not, as has been supposed, through the duodenum and the common biliary duct. 4. In the liver, glycerine produces a hypersecretion of the bile, and is, therefore, a cholagogue. 5. Administered during an attack of hepatic colic, in doses of from 20 to 30 grammes (300 to 450 graines) a day, in chloroform water, or in an alkaline medium, glycerine is superior to olive oil.

---

IODOZON IN PHTHISIS.—Iodozon (*Gazette Med. de Liege-Pharm. Centralbl., 1892, p. 468, Notes on New Remedies*, November, 1892) is the name applied to a liquid recommended as an efficient antiseptic, adapted to the treatment of wounds, etc., and as inhalation for phthisical patients. The drug is said to contain as a "basis a combination of iodine and ozone (?) as it occurs in the atmosphere, most especially on the sea-shore, where iodine and ozone are always present.

---

DIAPHTHERIN OR OXYCHINASEPTOL.—This new remedy, recently introduced into practical medicine, is chemically a combination of one molecule of ortho-phenol sulphonic acid (or aseptol) and two molecules of oxychinolin. It occurs in the form of a yellow powder; it is readily soluble in water, and at an elevated temperature decomposes into its components. With regard to its properties, Emmerich (*Therap. Monatshefte, 1892, p. 360; Notes on New Remedies*, November, 1892) considers diaphtherin the most powerful of antiseptics, and for practical purposes the most suitable. It may be classed with phenol, lysol, cresol, etc., and it is even more active than some of these. A 0.3% solution destroys staphylococcus pyogenes aureus in fifteen minutes; a 0.1% solution the bacillus pyocyaneus in forty-five minutes, and the cholera bacillus in ten minutes. A 0.2% solution destroys diphtheria bacillus in the same time. Its behavior toward fresh, undried spores is also very favorable. Notwithstanding its strong antiseptic properties, oxychinaseptol is relatively non-poisonous to animals.

---

THE TREATMENT OF BURNS BY THIOL.—Bidder (*Pharm. Centralbl.*, 1892, p. 466, Notes on New Remedies, November, 1892), highly recommends thiol in the treatment of burns. The author discards linseed oil and lime water, as the necessity for the frequent removal of this remedy irritates the affected parts and destroys the blisters, the latter protecting it from contact with micro-organisms. The method of application is simple. The affected and surrounding parts are painted with thiolum liquidum, diluted with an equal quantity of water, and then covered with a layer of cotton. This also relieves the pain in a short time.

---

PHENOLSALYL.—J. DeChristmas (*Annales de Institut Pasteur-Pharm-Zeit.*, No. 36, p. 434, 1892), gives the above name to an antiseptic mixture soluble in 25 parts of water and very readily so in glycerine. *Phenolsalyl* is said to almost equal corrosive sublimate in its antiseptic properties. The mixture is composed of the following:

| | |
|---|---|
| Carbolic acid | 9 parts |
| Salicylic acid | 1 part |
| Lactic acid | 2 parts |
| Menthol | 0.1 part |

---

THE INFLUENCE EXERCISED BY THE SIZE OF THE DOSE.—In an excellent contribution, bearing upon the action of medicines, Agustin M. Fernandez de Ibarra, of New York (*Gaceta Medico-Quirurgica*, November, 1892), treats of the above subject. A careless observer may, in glancing over the article, draw the erroneous conclusion that the author is defending the false doctrine of *similia similius curantur.* Fernandez de Ibarra contends that in the present status of medical science, the knowledge of botanical relations, so to speak, is not in itself sufficient for the medical observer to explain accurately the effects of plants, nor to be able to judge in regard to what effects will be produced by the amount of the drug given; and that, in consequence, we have to rely principally on clinical experience. [This perhaps may not be entirely correct; but, so far, it holds good in the majority of instances. Certainly, it may properly be held that no amount of experimental evidence can overthrow a clinical fact, but, on the other hand, clinical experience is not sufficient either in itself to explain the manner in which this or that medicament influences the organism.—D. C.] Although it is true that the effects of medicines may be divided into immediate and remote, and that these, exercising an influence upon a part of the body, upon an organ or a tissue, only increase or diminish the normal function of that tissue, organ or part of the body, without changing it or substituting it for another; yet, how such changes are brought about is, and will perhaps ever remain, unknown! For instance: Strychnine increases reflex action, but if the use of the medicament is continued for a long time the vitality of reflex action is exhausted, and is finally abolished (paralysis) if the employment of the drug is insisted upon. The author gives other good examples. He contends that good is produced when remedies are employed to relieve the same symptoms which they produce, and it would seem that the writer was drifting into the sea of the ridiculous doctrine of homeopathy. Such, however is not the case. Different effects are produced by the same drug, according to the dose used. Thus, aloes is aperient, cathartic or drastic, according the amount of the drug employed. Hemlock or conium, is a purge, a diuretic, an emetic, a hydragogue or an emmenagogue, under different doses; and so other examples could be cited to illustrate the subject. The author treats his theme in a masterly manner. From a thoughtful and practical study of the subject, he formulates the following: 1. It is a well-known fact that a teaspoonful of the syrup of ipecacuahna is sufficient to produce emesis; but it is no less a fact that 1 drop of the wine of ipecacuahna in a tablespoonful of water, administered every fifteen minutes, is of great service in checking frequent vomiting, especially that of pregnancy, and also that occurring in subacute gastritis, and even in that which, accompanied with diarrhœa, is seen in children. 2. Tartar emetic in doses of 5 milligrammes (1-12 of a grain) is a diaphoretic and at the same time a slight cardiac depressant; while in amounts of 10 centigrammes (1-12 grains) is an active emetic. 3. One milligramme (1-64 of a grain) of calomel, given every hour, during 10 or 12 consecutive hours, relieves headache of syphilitic origin, a symptom generally occurring at night. Undoubtedly, this substance, administered in such quantities, is wholly absorbed and thus carried to the brain by the current of the circulation. 4. It is well-known that cantharides, in large doses, produces intense inflammation of the urinary tract; but it is also a fact that one single drop of the tincture of the remedy, taken every hour in a half-glassful of water, relieves many case of true vesical catarrh. 5. Infantile diarrhœa, complicated with a slight intestinal inflammation and mucous discharges (without the disorder being a true dysentrey), castor oil, in doses of 3 drops, administered in sweetened water and a little gum acacia, is an excellent remedy. 6. In cases of orchitis and epididymitis great relief is obtained in a short time, from the administration every hour of 2 drops of the tincture of pulsatilla in a little water. 7. No one denies that the fluid extract of ergot is highly useful in the treatment of excessive menstruation. On the other hand, it is a fact (although apparently paradoxical and perhaps not generally known) that the same medicament, in doses of 1 drop every half-an-hour, is quite efficient in cases of amenorrhœa not dependent upon anæmia. 8. Balsam of copaiba in large quantities produces urticaria, and yet, 1 drop of this remedy, ingested every hour, often cures cases of that cutaneous affection. 9. Fowler's solution, administered in doses of 1-2 a drop every half-an-hour, during 3 or 4 consecutive hours, will relieve the vomiting occurring after a debauchery, and is similarly useful in the vomiting of pregnancy. 10. One gramme (16 minims) of a solution of the sulphate of atrophine, of the strength of 1-200 of a grain in a glassful of water, and of which the dose shall be a teaspoonful every hour, produces great relief in cases of pseudo-croupous disorders of reflex origin which are often observed in children. In such instances, however, when the full physiological effects of the drug begin to manifest themselves, the intervals between the doses should be increased.

### The Active Principles of Cod Liver Oil.

The remarkable studies of M. M. Gautier and Mourgues on the alkaloids of Cod Liver Oil, show us definitely the nature of the principles to which are due, to a very great extent, its medicinal properties.

The physiological experiments made by these authorities on animals prove that the alkaloids referred to act:

1st. As stimulants of nutrition and of the circulation.

2nd. As diuretics.

In presence of such remarkable results explaining the therapeutic action of the oil, I have thought it possible to utilize in medicine the alkaloids themselves; besides it appeared to me interesting to inquire if the effects observed by M. M. Gautier and Mourgues, in their experimentation on animals, and especially its action as a stimulant to the appetite and diuresis, were noticeable when exhibited in a human being.

Guided by this idea, I prepared some of these same alkaloids, but in the present case I have not attempted to isolate them, and I have administered, therefore, the whole of the active principles of Cod Liver Oil as a medicinal unit.*

The dose administered by the mouth to normal subjects in 24 hours, varied from 15 to 25 centigrammes.

The analysis of the urine made before and after the administration of these alkaloids showed that—

1st. The volume of urine voided during the 24 hours, as well as the amount of urea, was considerably increased.

2nd. That it acted as a powerful stimulant to the intra-organic oxidization, a fact already formulated in the conclusions of the original work.

From a clinical point of view the following are some of the results obtained on treating a number of patients with the active principles of Cod Liver Oil:

1st. Five young women, with vague pains, loss of appetite, progressive decrease of strength, neur-asthenia. The effects in the first place were increase of appetite, return of strength, with loss of the painful symptoms referred to. Three of them who had not menstruated for a considerable period were relieved of the suppression in a short time after beginning the treatment.

2nd. In the case of two children who were suffering from malnutrition, the appetite promptly returned in a few days.

3rd. Three patients who were afflicted with severe eczematous eruptions at each menstrual period, were cured of this trouble.

4th. In two cases of bronchial catarrh in old patients, the alkaloids produced the well-known effect of Cod Liver Oil, and were administered with advantage and perfectly tolerated.

These observations show that the active principles of Cod Livei Oil are of undoubted value as therapeutic aids where the oil is indicated.—*Translated from the French, by Dr. F. S. Mason, N. Y.*

---

*M. Chapoteaut, in 1885, was the first to demonstrate that, apart from the fat considered as an assimilable fat, there existed, in variable proportions, a number of alkaloids, etc., and these he removed from Cod Liver Oil in the form of Morrhuol, representing all its active principles.

